# Design of a New α-1-*C*-Alkyl-DAB Derivative Acting as a Pharmacological Chaperone for β-Glucocerebrosidase Using Ligand Docking and Molecular Dynamics Simulation

**DOI:** 10.3390/molecules23102683

**Published:** 2018-10-18

**Authors:** Izumi Nakagome, Atsushi Kato, Noriyuki Yamaotsu, Tomoki Yoshida, Shin-ichiro Ozawa, Isao Adachi, Shuichi Hirono

**Affiliations:** 1 School of Pharmacy, Kitasato University, 5-9-1 Shirokane, Minato-ku, Tokyo 108-8641, Japan; yamaotsun@pharm.kitasato-u.ac.jp (N.Y.); yoshidat@pharm.kitasato-u.ac.jp (T.Y.); ozawas@pharm.kitasato-u.ac.jp (S.-i.O.); 2 Department of Hospital Pharmacy, University of Toyama, 2630 Sugitani, Toyama 930-0194, Japan; kato@med.u-toyama.ac.jp (A.K.); adachi@med.u-toyama.ac.jp (I.A.)

**Keywords:** pharmacological chaperone, Gaucher disease, ligand docking, molecular dynamics, drug design

## Abstract

Some point mutations in β-glucocerebrosidase cause either improper folding or instability of this protein, resulting in Gaucher disease. Pharmacological chaperones bind to the mutant enzyme and stabilize this enzyme; thus, pharmacological chaperone therapy was proposed as a potential treatment for Gaucher disease. The binding affinities of α-1-*C*-alkyl 1,4-dideoxy-1,4-imino-d-arabinitol (DAB) derivatives, which act as pharmacological chaperones for β-glucocerebrosidase, abruptly increased upon elongation of their alkyl chain. In this study, the primary causes of such an increase in binding affinity were analyzed using protein–ligand docking and molecular dynamics simulations. We found that the activity cliff between α-1-*C*-heptyl-DAB and α-1-*C*-octyl-DAB was due to the shape and size of the hydrophobic binding site accommodating the alkyl chains, and that the interaction with this hydrophobic site controlled the binding affinity of the ligands well. Furthermore, based on the aromatic/hydrophobic properties of the binding site, a 7-(tetralin-2-yl)-heptyl-DAB compound was designed and synthesized. This compound had significantly enhanced activity. The design strategy in consideration of aromatic interactions in the hydrophobic pocket was useful for generating effective pharmacological chaperones for the treatment of Gaucher disease.

## 1. Introduction

**Figure 1:**
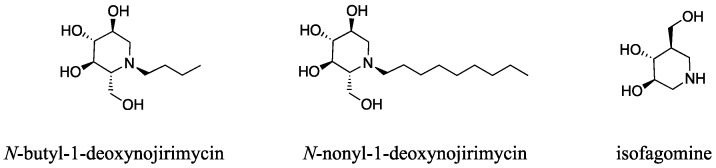
Known pharmacological chaperones for β-glucocerebrosidase.

β-Glucocerebrosidase (EC 3.2.1.45) is a lysosomal enzyme responsible for the metabolism of glucosylceramides. Mutations of the gene encoding this enzyme are the cause of Gaucher disease, one of the most common lysosomal storage disorders [1,2]. Some mutations may lead to either an improper folding of the protein or instability of its native conformation; as a result, the translocation of this enzyme from the endoplasmic reticulum to the lysosome is reduced [3]. The clinical manifestations of Gaucher disease include hypertrophy of the spleen or liver, anemia, bone lesions accompanied by pain, and, in some cases, central nervous system impairment [4,5]. Recently, pharmacological chaperone therapy was proposed as a treatment for Gaucher disease. This therapeutic strategy is based on the concept that reversible competitive inhibitors can stabilize the three-dimensional conformations of mutated enzymes, thereby assisting the transport of these enzymes to the native cellular compartment [6,7]. It was reported that *N*-butyl-1-deoxynojirimycin, *N*-nonyl-1-deoxynojirimycin, and isofagomine can act as pharmacological chaperones (Figure 1) [8,9].

The parent iminosugar of these molecules is similar to d-glucose, which is a component of the substrate. This structural similarity leads to a strong affinity for β-glucocerebrosidase, while these compounds also show inhibitory activity against α-glucosidase, which might be linked to their side effects [10,11,12]. Recently, isofagomine entered clinical phase II trials as a potential pharmacological chaperone. However, the anticipated biological effect was not observed despite the formation of a favorable hydrogen-bonding network within the binding pocket. This reason was considered to be a weak hydrophobic interaction [13,14]. From this background, isofagomine analogs bearing an alkyl chain at the C6 position were designed and these compounds displayed an improvement in half maximal inhibitory concentration (IC_50_) [5]. These results suggested the presence of a hydrophobic pocket close to the ligand-binding site and the importance of the hydrophobic moiety of the ligand. On the other hand, isofagomine analogs bearing *N*-alkyl chains were less potent inhibitors [5]. Therefore, knowledge about the physicochemical properties of the hydrophobic site accommodating the alkyl chains is important for the development of new pharmacological chaperones. However, so far, there are only a few studies about the hydrophobic ligand-binding sites of β-glucocerebrosidase.

**Figure 2:**
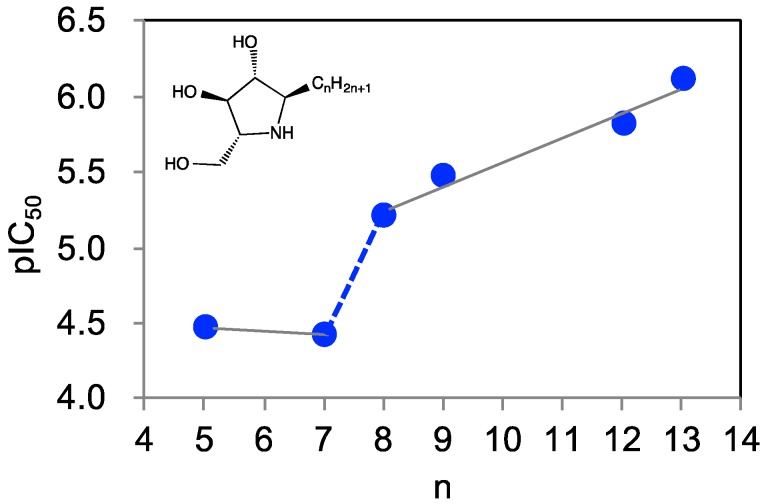
The graph of activity (negative log of half maximal inhibitory concentration (pIC_50_)) of α-1-*C*-alkyl 1,4-dideoxy-1,4-imino-d-arabinitol (DAB) derivatives against human β-glucocerebrosidase (n: alkyl chain length).

We previously designed a series of pyrrolidine-based pharmacological chaperones, namely α-1-*C*-alkylated 1,4-dideoxy-1,4-imino-d-arabinitol derivatives (α-1-*C*-alkyl-DAB derivatives) [15]. These α-1-*C*-alkyl-DAB derivatives displayed potent and selective inhibition of β-glucocerebrosidase. Furthermore, these compounds improved the hydrolytic activity of the mutated protein, as well as its translocation to the lysosome. In the process of optimizing α-1-*C*-alkyl-DAB derivatives, we found that their affinities for β-glucocerebrosidase drastically increased with elongation of the alkyl chain (Figure 2).

α-1-*C*-Butyl-DAB exhibited no affinity for β-glucocerebrosidase even at a concentration of 1000 μM, whereas inhibitory activity was expressed by the introduction of a pentyl or heptyl chain at C1 of DAB (IC_50_ values of 34 and 38 μM, respectively). Furthermore, the extension of the length of the α-1-*C*-alkyl chain tended to improve inhibition potency accordingly. α-1-*C*-Octyl-DAB and α-1-*C*-nonyl-DAB showed potent inhibition activities (IC_50_ values of 6.2 and 3.3 μM, respectively). The most potent inhibitor α-1-*C*-tridecyl-DAB displayed a remarkable IC_50_ value of 0.77 μM, which is 44-fold more potent relative to α-1-*C*-pentyl-DAB. In particular, the presence of a so-called “activity cliff” was observed between α-1-*C*-heptyl-DAB and α-1-*C*-octyl-DAB. We also reported the inhibitory activities of these compounds correlated with chaperone activities. These results clearly suggested that there is a minimum 1-*C*-alkyl chain length requirement for achieving inhibition of β-glucocerebrosidase. Furthermore, analysis of the difference in the binding mode of α-1-*C*-heptyl-DAB and α-1-*C*-octyl-DAB led to an understanding of the shape of the hydrophobic site accommodating the alkyl chain on this protein, and elucidated the amino-acid residues that are critical to the binding of the ligand. In this study, we elucidated the primary factors responsible for the drastic increase in binding affinity in association with alkyl chain elongation using computational ligand docking and molecular dynamics (MD) simulations. Furthermore, we designed and synthesized a new compound utilizing the characteristics of the hydrophobic site accommodating the alkyl chains.

## 2. Results

### 2.1. Docking Results

The resulting structures from an induced-fit docking were ranked by an induced-fit docking score. The top 10 structures were energy-minimized; then, the interaction energies were calculated. The three lowest-interaction-energy structures were selected and observed (Table S1, Figure S1). In the case of the complex structures of α-1-*C*-octyl-DAB, α-1-*C*-nonyl-DAB, and α-1-*C*-dodecyl-DAB, the orientations of the pyrrolidine groups were similar and the alkyl chains were accommodated in the hydrophobic groove located between loop 1 (amino-acid residues 311–319) and loop 2 (amino-acid residues 342–354). On the other hand, in the case of α-1-*C*-pentyl-DAB and α-1-*C*-heptyl-DAB, the alkyl chains were accommodated in a vestibular pocket near the hydrophobic groove. In the case of α-1-*C*-tridecyl-DAB, two types of the complex structure were generated. The orientation of the alkyl chain of the lowest-interaction-energy structure (n13A) was different from that of the other compound, and the orientation of the alkyl chain of the third lowest-interaction-energy structure (n13B) was the same as that of the other compound. Therefore, the complex structure with α-1-*C*-tridecyl-DAB was decided from the results of MD simulation described below. MD simulations were performed in aqueous solution in order to refine the complex structures and analyze the dynamic properties of the α-1-*C*-alkyl-DAB derivatives. The most stable structures from among the three complex structures were used as initial structures in the simulations, except for α-1-*C*-tridecyl-DAB, in which case, two types of complex structures (n13A and n13B) were used as initial structures.

### 2.2. Results of MD Calculations

**Figure 3:**
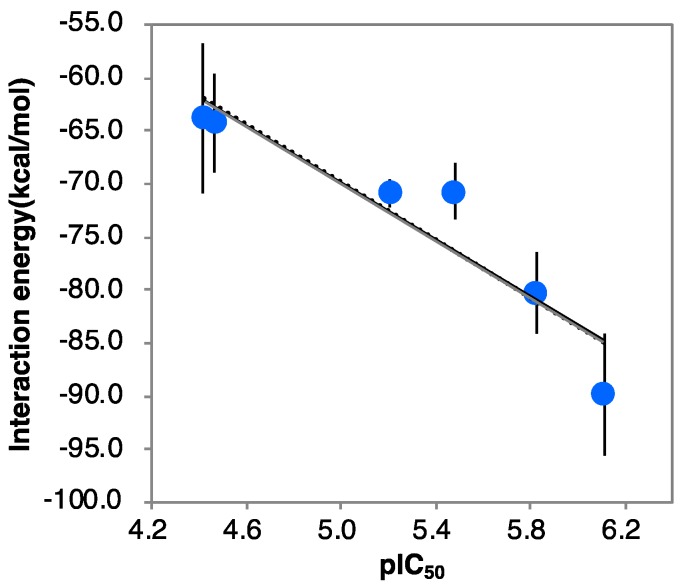
The correlation between pIC_50_ values and interaction energies (*r*^2^ = 0.87). Error bars indicate standard deviation.

The root-mean-square deviations (RMSDs) from the initial structures were calculated for each MD trajectory (Figure S2). Then, 42 complex structures were extracted from 5 to 6 ns where the RMSD was stable. The interaction energies were calculated for 42 complex structures using the OPLS2005 force field using molecular mechanics/Generalized Born solvent accessible surface area (MM/GBSA) conditions after energy minimization. The results for α-1-*C*-tridecyl-DAB showed that the average interaction energy of n13B was lower than that of n13A (−89.80 vs. −81.22 kcal/mol, respectively). These results showed that n13B was suitable as the complex structure of α-1-*C*-tridecyl-DAB with β-glucocerebrosidase. The average interaction energies of the six α-1-*C*-alkyl-DAB derivatives decreased as alkyl chain length increased (Table S2). A high correlation of 0.87 was observed between the average interaction energies and experimental negative log IC_50_ (pIC_50_) values (Figure 3).

**Figure 4:**
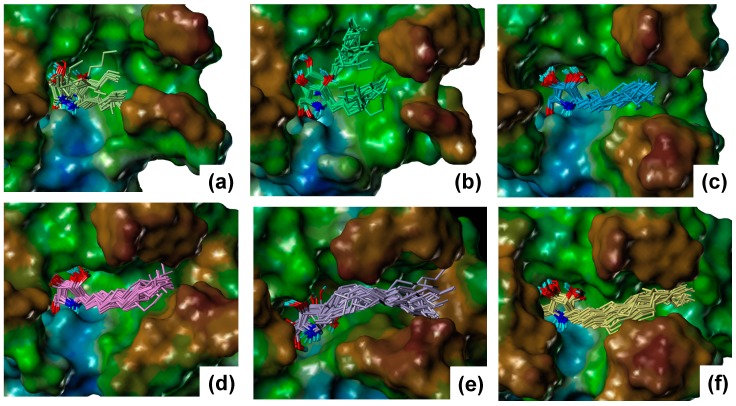
The binding poses of the α-1-*C*-alkyl-DAB derivatives extracted from the molecular dynamics (MD) trajectory: (**a**) α-1-*C*-pentyl-DAB, (**b**) α-1-*C*-heptyl-DAB, (**c**) α-1-*C*-octyl-DAB, (**d**) α-1-*C*-nonyl-DAB, (**e**) α-1-*C*-dodecyl-DAB, and (**f**) α-1-*C*-tridecyl-DAB. The molecular surface of β-glucocerebrosidase is rendered using a lipophilic potential (drawn by Sybyl-X 2.1.1 Certara, LP, Princeton, NJ, USA).

This result suggests that the predicted structures are reliable. Snapshots of the MD simulations are shown in Figure 4.

**Figure 5:**
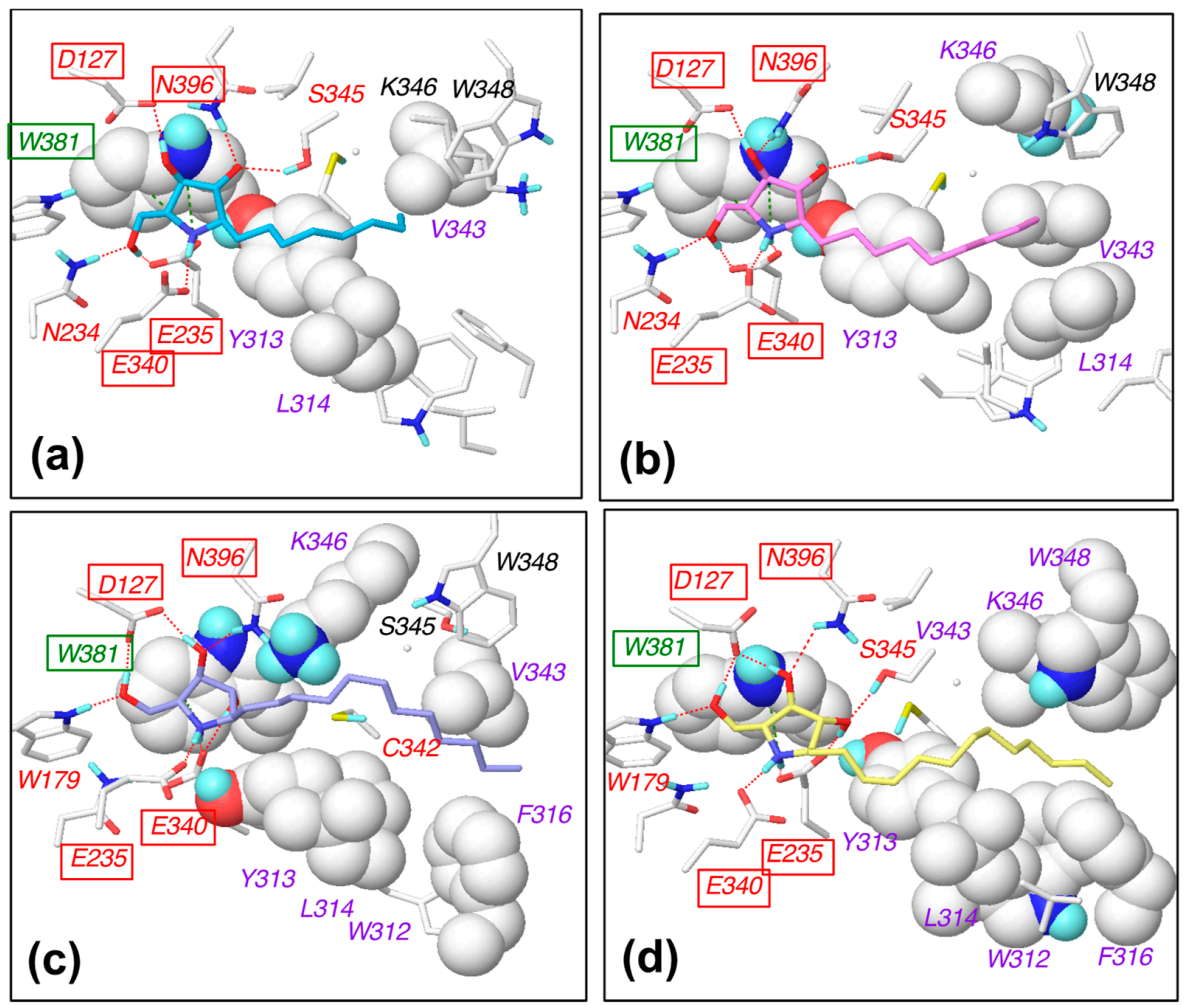
Schematic diagram of the interactions between β-glucocerebrosidase and (**a**) α-1-*C*-octyl-DAB, (**b**) α-1-*C*-nonyl-DAB, (**c**) α-1-*C*-dodecyl-DAB, and (**d**) α-1-*C*-tridecyl-DAB. The labels of hydrogen-bonding amino-acid residues, cation–π interacting amino-acid residues, and hydrophobically interacting amino-acid residues are colored in red, green, and purple, respectively. The labels of commonly interacting amino acids are boxed.

For α-1-*C*-pentyl-DAB and α-1-*C*-heptyl-DAB, multiple ligand orientations were found; these ligands moved dynamically within the vestibular pocket. For α-1-*C*-heptyl-DAB, two types of interactions of the alkyl chain were observed. In one type, the alkyl chain was oriented to the groove between loop 1 and loop 2, while, in the other type, the alkyl chain was oriented to the groove between loop 2 and loop 3. The results of α-1-*C*-pentyl-DAB and α-1-*C*-heptyl-DAB showed that multiple ligand orientations were generated due to their short alkyl chains, as well as the size of the vestibular pocket. In other words, these ligands were unable to form stable interactions because of a deficit of hydrophobic interactions; hence, their binding activities probably decreased. The compounds with long alkyl chains, such as α-1-*C*-octyl-DAB, α-1-*C*-nonyl-DAB, α-1-*C*-dodecyl-DAB, and α-1-*C*-tridecyl-DAB, were bound to the hydrophobic groove located between loop 1 and loop 2. These results suggested that the “activity cliff” between α-1-*C*-heptyl-DAB and α-1-*C*-octyl-DAB was due to the difference in shape of the binding site. This is the first report that the size and shape of this hydrophobic site in the β-glucocerebrosidase strictly controls the binding affinity of the ligands. Next, the interactions of α-1-*C*-octyl-DAB, α-1-*C*-nonyl-DAB, α-1-*C*-dodecyl-DAB, and α-1-*C*-tridecyl-DAB were observed (Figure 5).

The hydroxyl groups in the pyrrolidine ring formed hydrogen bonds with the Asp127, Glu235, Glu340, and Asn396 residues of β-glucocerebrosidase in all complexes. The nitrogen atom in the pyrrolidine ring formed a cation–π interaction with Trp381. Van der Waals interactions were observed between alkyl chains and Tyr313, Leu314, and Val343. Furthermore, alkyl chains of α-1-*C*-dodecyl-DAB and α-1-*C*-tridecyl-DAB formed van der Waals interactions with Trp312 and Phe316. The orientations of the pyrrolidine rings were stable in the MD simulations. We considered that these complexes were stabilized by the favorable hydrophobic interaction between the hydrophobic groove and the longer alkyl chain, in addition to the hydrogen-bonding interaction with the pyrrolidine ring.

### 2.3. Fluctuation of Loop 2

**Figure 6:**
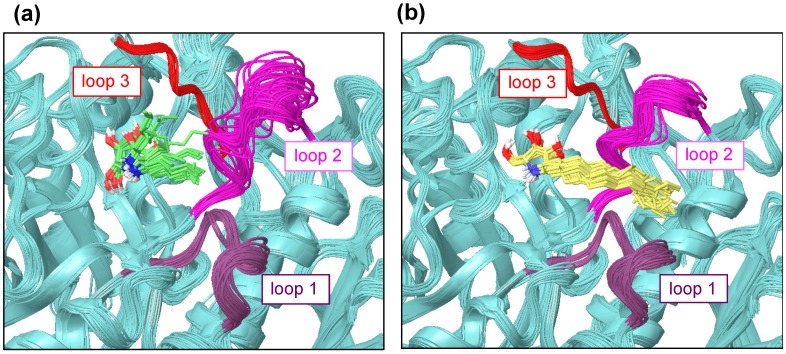
The loop fluctuations in β-glucocerebrosidase with (**a**) α-1-*C*-pentyl-DAB (green) and (**b**) α-1-*C*-tridecyl-DAB (yellow). Loop 1 is colored in purple (amino acids 311–319); loop 2 is colored in magenta (amino acids 342–354); loop 3 is colored in red (amino acids 394–399).

The ligand-binding site of β-glucocerebrosidase is surrounded by three loops [16]. The multiple crystal structures show that these three loops are very flexible. The multiple ligand orientations were observed in the binding site featuring α-1-*C*-alkyl-DAB derivatives with short alkyl chains from the MD trajectory. In spite of the limited MD time scale, a fluctuation of loop 2 was observed for α-1-*C*-pentyl-DAB, whereas such fluctuation was small for α-1-*C*-tridecyl-DAB (Figure 6).

The interaction between the alkyl chain of α-1-*C*-tridecyl-DAB and the hydrophobic region of β-glucocerebrosidase might have reduced the fluctuation of loop 2 near the active site.

### 2.4. Modeling of a Complex Structure of β-Glucocerebrosidase with Glucosylceramide

**Figure 7:**
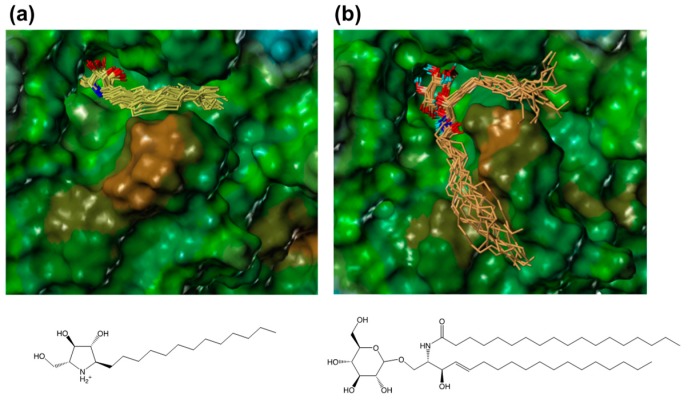
The binding poses of (**a**) α-1-*C*-tridecyl-DAB (yellow) and (**b**) glucosylceramide (orange) extracted from the MD trajectory. The molecular surface of β-glucocerebrosidase is rendered using a lipophilic potential (drawn by Sybyl-X 2.1.1).

In order to compare the interactions of α-1*C*-alkyl-DAB derivatives with the interaction of the natural substrate, we attempted to model a complex structure of β-glucocerebrosidase with glucosylceramide. Glucosylceramide consists of a single glucose residue and a lipid moiety called ceramide that comprises a long-chain amino alcohol (sphingosine) and a fatty acid. The complex structure of glucosylceramide with β-glucocerebrosidase was modeled using a similar strategy as that employed for α-1-*C*-alkyl-DAB derivatives. The model showed that the two long chains of glucosylceramide individually bound to different hydrophobic grooves (Figure 7).

The sphingosine moiety of glucosylceramide bound to the hydrophobic groove located between loop 1 and loop 2, which accommodates the alkyl chain of α-1-*C*-alkyl-DAB derivatives, while the lipid moiety bound to another groove located between loop 1 and another loop (amino-acid residues 233–250). The latter binding site included Gln284, Leu314, His290, Leu286, Leu287, Ala318, and Pro319, and tended to be slightly hydrophilic. It was found that the long alkyl chains of the α-1-*C*-alkylated-DAB derivatives were accommodated to the more hydrophobic groove among the two grooves accommodating glucosylceramide.

### 2.5. Design of 7-(Tetralin-2-yl)-heptyl-DAB

**Figure 8:**
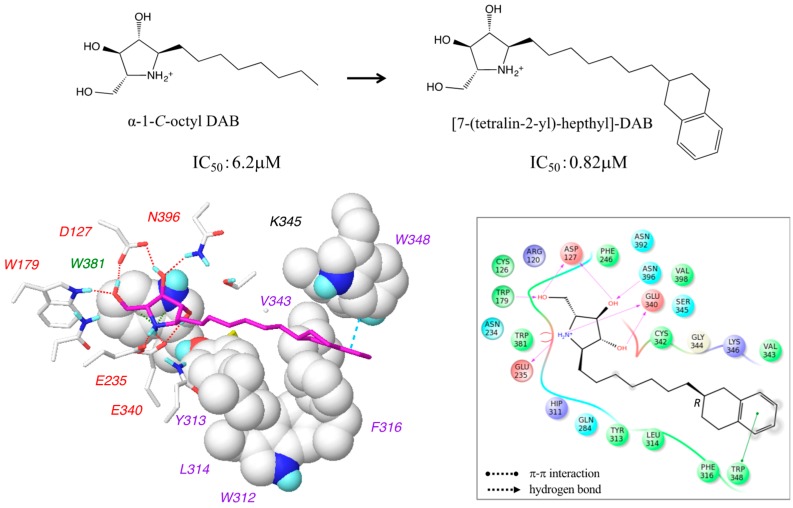
The docking model and the interaction mode of 7-(tetralin-2-yl)-heptyl-DAB complexed with β-glucocerebrosidase. The experimental activity of 7-(tetralin-2-yl)-heptyl-DAB was measured using the diastereomeric mixture. The interaction diagram is displayed using the *R*-isomer with a slightly favorable interaction energy.

We found that the hydrophobic groove accommodating the alkyl chains of the α-1-*C*-alkyl-DAB derivatives was rich in aromatic amino acids, such as Trp312, Tyr313, Phe316, and Trp348. Therefore, we synthesized 7-(tetralin-2-yl)-heptyl-DAB by introducing an aromatic tetralin group into the alkyl chain of α-1-*C*-octyl-DAB. Despite the diastereomeric mixture, the IC_50_ of 7-(tetralin-2-yl)-heptyl-DAB was about eightfold higher compared to its parent compound, α-1-*C*-octyl-DAB (0.82 µM vs. 6.2 µM, respectively). Accordingly, we built the complex structure model of 7-(tetralin-2-yl)-heptyl-DAB with β-glucocerebrosidase. 7-(Tetralin-2-yl)-heptyl-DAB has a chiral center at the C7 carbon. The complex structures of β-glucocerebrosidase with 7-(tetralin-2-yl)-heptyl-DAB were built for both the *R*- and *S*-isomers. These complex structures presented similar binding modes between *R*- and *S*-isomers. The interaction energies calculated from the complex structures showed that both diastereomers may possibly have affinity for β-glucocerebrosidase. The interaction diagram was displayed using the *R*-isomer with a slightly favorable interaction energy (Figure 8).

The van der Waals interactions were formed between the tetralin group and Trp312 and Phe316, while retaining the hydrogen-bond network at the pyrrolidine ring. Additionally, a π–π interaction was introduced between the tetralin group and Trp348. The increase in activity of the designed compound and the predicted binding mode supported the interaction modes between α-1-*C*-alkyl-DAB derivatives and β-glucocerebrosidase predicted in this study. The design strategy in consideration of aromatic interactions in the hydrophobic pocket is a new point of view for the development of new effective pharmacological chaperones for the treatment of Gaucher disease.

## 3. Discussion

The activity of the pharmacological chaperone for β-glucocerebrosidase includes many factors: the binding of the chaperone to the enzyme, the stabilization of the enzyme, the transport of the enzyme to the lysosome, the replacement with the substrate in the lysosome, the protein–protein interactions with other proteins (such as sapocin C or phospholipids) in the lysosome, and the cell permeability of the chaperone. This study focused on the binding of the chaperones to β-glucocerebrosidase. Herein, we highlighted the importance of the interaction with the hydrophobic groove of β-glucocerebrosidase for pharmacological chaperones, using computational ligand docking and MD simulations. In particular, we found that the size and shape of this hydrophobic site, composed of the vestibular pocket and the hydrophobic groove, strictly controlled the binding affinity of the ligands. Additionally, we found the hydrophobic groove is rich in aromatic amino acid residues; hence, we designed and synthesized 7-(tetralin-2-yl)-heptyl-DAB. The binding affinity of this compound turned out to be significantly enhanced, suggesting that the activity of the designed compound may be further increased by adding an aromatic interaction to the hydrophobic interaction. The design of 7-(tetralin-2-yl)-heptyl-DAB, focusing on the aromatic/hydrophobic interactions between chaperones and β-glucocerebrosidase, may lead to the development of effective pharmacological chaperones for the treatment of the Gaucher disease.

## 4. Materials and Methods

### 4.1. Docking Analysis

The crystal structure of a complex of β-glucocerebrosidase with isofagomine (Protein Data Bank identifier: 2NSX) was obtained from the Protein Data Bank (PDB), as this crystal structure was determined at pH 5.5 reflecting the lysosome’s acidic environment [17]. The docking analysis was performed according to a previously reported method [18]. Since the orientation of the Asn396 side chain differed between the B and D chains to which isofagomine binds, an induced-fit docking protocol (version 3.1, Schrödinger, LLC, New York, NY, USA) was used [19]. The preparation of the protein structure was performed using Maestro Protein Preparation Wizard (version 10.3, Schrödinger, LLC, New York, NY, USA) using the B chain. Water molecules were removed from the crystal structure and hydrogen atoms were added in an acidic environment (pH 5.5) for consistency with the experimental conditions. The docking site was defined as an enclosing box (26 × 26 × 26 Å) centered at the centroid of the co-crystallized ligand. The three-dimensional structures of the α-1-*C*-alkyl-DAB derivatives (alkyl chain lengths of 5, 7, 8, 9, 12, and 13 carbon atoms) were built using Chem3D (PerkinElmer, Inc., Waltham, MA, USA), hydrogens were added using LigPrep (version 3.5, Schrödinger, LLC, New York, NY, USA) in Maestro, and then, multiple conformations were generated using ConfGen (version 3.3, Schrödinger, LLC, New York, NY, USA) in Maestro. LigPrep can produce a number of structures with various ionization states and ring conformations from the input structure. ConfGen can rapidly and systematically explore broad conformational space. Induced-fit docking analyses were performed under standard precision (SP) mode. For the first step of the induced-fit docking protocol, Asn396 was temporarily mutated to alanine, and the analysis was performed using a soft potential, i.e., the van der Waals radius scaling for ligand and protein was 0.5 and 0.7, respectively. In the second step, Ala396 was reverted to asparagine, and the amino-acid residues located within 5 Å of the docked ligand were refined to adjust their side chains. In the final step, the ligand was again docked to the induced-fit receptor structure, and the resulting structures were ranked using an induced-fit docking score. Each of the top 10 structures was energy-minimized using the OPLS2005 force field and 0.05 kcal/mol/Å of convergence with a distance-dependent dielectric constant (ε = 4). Subsequently, the interaction energies were calculated with a distance-dependent dielectric constant (ε = 4), and the three lowest-energy complex structures were selected.

### 4.2. MD Calculations

MD calculations were performed for the complex structures of the α-1-*C*-alkyl-DAB derivatives with β-glucocerebrosidase using Desmond (version 4.3, Schrödinger, LLC, New York, NY, USA) [20,21,22] with an OPLS_2005 force field. The system was solvated in a cubic simulation box using a simple point charge (SPC) water model with periodic boundary conditions. The net charge of the system was neutralized using the particle mesh Ewald method by addition of sodium and chloride ions. Next, the energy minimization of the system was carried out with a maximum of 20,000 cycles with 0.05 kcal/mol/Å of convergence. The MD simulations were performed for 6 ns at 310 K and 1 atm using two initial rate conditions. Snapshots of each system were sampled from the MD trajectory at an interval of 48 ps. All calculations were performed using the Schrödinger Suite 2015-3 (Schrödinger, LLC, New York, NY, USA).

### 4.3. Enzyme Inhibition Assay

The inhibitory activity against β-glucocerebrosidase was measured using Cerezyme (Genzyme; Boston, MA, USA) as an enzyme source and 4-methylumbelliferyl-β-d-glucopyranoside (Sigma-Aldrich Co., St. Louis, MO, USA) as the substrate. The reaction mixture consisted of 100 mM McIlvaine buffer (pH 5.2), 0.25% sodium taurocholate, 0.1% Triton X-100 (Nacalai Tesque Inc., Kyoto, Japan), and the appropriate amount of enzyme. The reaction mixture was preincubated at 0 °C for 45 min; then 3 mM substrate solution was added, followed by incubation at 37 °C for 30 min. The reaction was stopped upon addition of 1.6 mL of a 400 mM glycine–NaOH solution (pH 10.6). The released 4-methylumbelliferone was measured (excitation wavelength = 362 nm; emission wavelength = 450 nm) using a F-4500 fluorescence spectrophotometer (Hitachi, Tokyo, Japan).

## Supplementary Materials

The following are available online: Figure S1: Three poses obtained from induced-fit docking; Figure S2: Variations of root-mean-square deviations from the initial structure in the process of molecular dynamics simulation; Table S1: Induced-fit docking score (IFDScore) and ΔE of three poses obtained from induced-fit docking; Table S2: IC_50_ (µM), pIC_50_, and calculated interaction energies of α-1-*C*-alkyl-DAB derivatives against human β-glucocerebrosidase.
