# Supplementary material for: Design of a New α-1-C-Alkyl-DAB Derivative Acting as a Pharmacological Chaperone for β-Glucocerebrosidase Using Ligand Docking and Molecular Dynamics Simulation
Source: Molecules. 2018 Oct 18;23(10):2683. doi: 10.3390/molecules23102683 (PMC6222826; doi:10.3390/molecules23102683)
Supplement: Supplementary file 1 [file molecules-23-02683-s001.pdf]

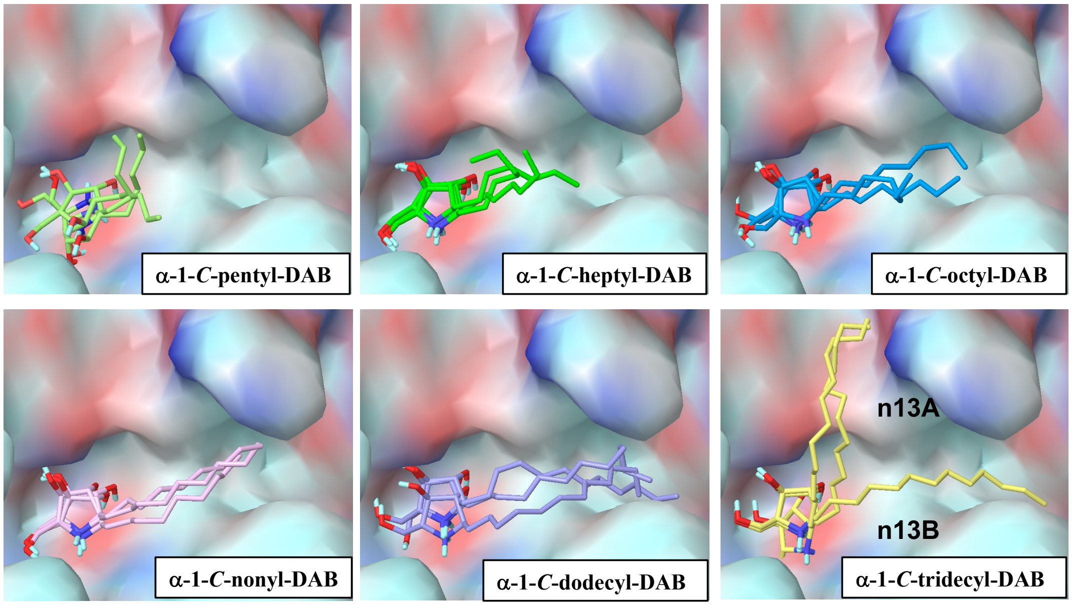

Figure S1. Three poses obtained from Induced fit docking. Images were produced using Maestro. The molecular surface is indicated by the atom color.

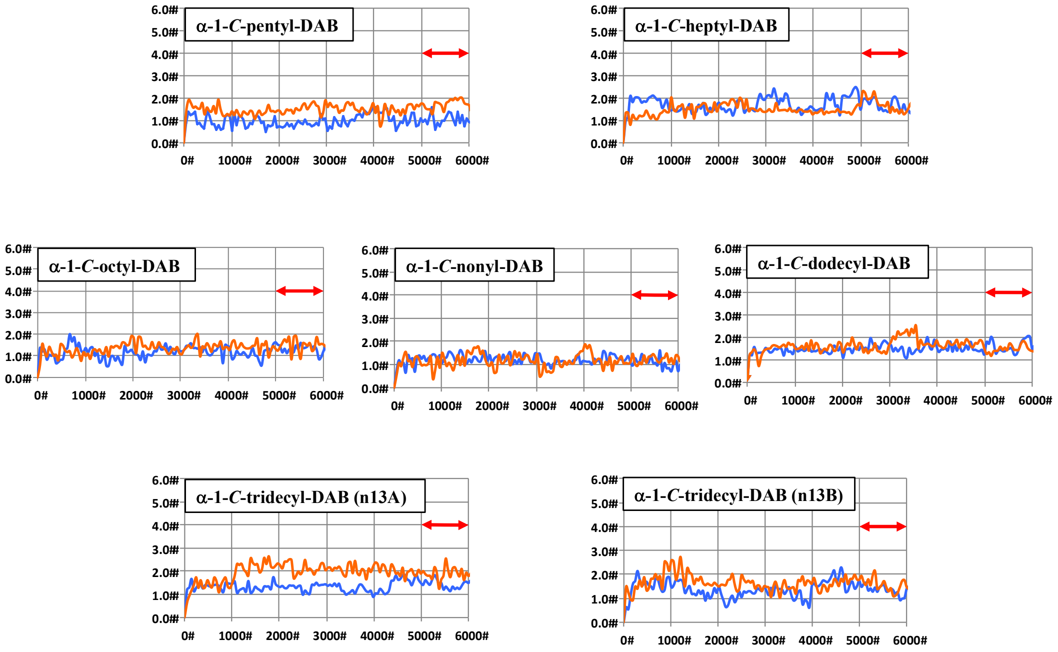

Figure S2. Variations of root-mean-square deviation from the initial structure in the process of molecular dynamics simulation (blue: MD1, orange: MD2).

Table S1. Induced Fit Docking score (IFDScore) and ΔE of three poses obtained from Induced fit docking.

	No.	IFDScore	ΔE (kcal/mol)
α-1-*C*-pentyl-DAB	1	-889.54	-47.51
	2	-889.25	-46.66
	3	-889.19	-45.50
α-1-*C*-heptyl-DAB	1	-889.06	-54.96
	2	-889.38	-54.92
	3	-889.75	-53.64
α-1-*C*-octyl-DAB	1	-889.80	-58.74
	2	-889.85	-57.17
	3	-889.39	-57.15
α-1-*C*-nonyl-DAB	1	-890.60	-58.55
	2	-890.58	-57.96
	3	-890.28	-57.91
α-1-*C*-dodecyl-DAB	1	-893.29	-68.01
	2	-893.37	-66.52
	3	-893.91	-66.02
α-1-*C*-tridecyl-DAB	1	-894.73	-70.01 (type A)
	2	-894.63	-69.83 (type A)
	3	-895.86	-67.90 (type B)

Table S2. IC_{50} (μM), pIC_{50} and the calculated interaction energy of α-1-*C*-alkyl-DAB derivatives against human β-glucocerebrosidase.

Compounds	IC_{50}	pIC_{50}	ΔE (kcal/mol)
α-1-*C*-pentyl-DAB	34	4.47	-64.17
α-1-*C*-heptyl-DAB	38	4.42	-63.76
α-1-*C*-octyl-DAB	6.2	5.21	-70.83
α-1-*C*-nonyl-DAB	3.3	5.48	-70.73
α-1-*C*-dodecyl-DAB	1.5	5.82	-80.26
α-1-*C*-tridecyl-DAB	0.77	6.11	-89.80
